# Light Enhances Survival of *Dinoroseobacter shibae* during Long-Term Starvation

**DOI:** 10.1371/journal.pone.0083960

**Published:** 2013-12-30

**Authors:** Maya Soora, Heribert Cypionka

**Affiliations:** Institute for Chemistry and Biology of the Marine Environment (ICBM), Carl-von-Ossietzky University of Oldenburg, Oldenburg, Germany; Laurentian University, Canada

## Abstract

Aerobic anoxygenic phototrophs (AAPs) as being photoheterotrophs require organic substrates for growth and use light as a supplementary energy source under oxic conditions. We hypothesized that AAPs benefit from light particularly under carbon and electron donor limitation. The effect of light was determined in long-term starvation experiments with *Dinoroseobacter shibae* DFL 12^T^ in both complex marine broth and defined minimal medium with succinate as the sole carbon source. The cells were starved over six months under three conditions: continuous darkness (DD), continuous light (LL), and light/dark cycle (LD, 12 h/12 h, 12 µmol photons m^−2^ s^−1^). LD starvation at low light intensity resulted in 10-fold higher total cell and viable counts, and higher bacteriochlorophyll *a* and polyhydroxyalkanoate contents. This coincided with better physiological fitness as determined by respiration rates, proton translocation and ATP concentrations. In contrast, LD starvation at high light intensity (>22 µmol photons m^−2^ s^−1^, LD conditions) resulted in decreasing cell survival rates but increasing carotenoid concentrations, indicating a photo-protective response. Cells grown in complex medium survived longer starvation (more than 20 weeks) than those grown in minimal medium. Our experiments show that *D. shibae* benefits from the light and dark cycle, particularly during starvation.

## Introduction

Aerobic anoxygenic phototrophs (AAPs) are widespread in marine habitats [1]–[3] but also occur in brackish [4]–[5] and fresh waters [6]–[7], saline lakes [8], soil [9], and hot springs [10]. Environmental studies based on infrared microscopy [11], pigment extraction [12], or sequencing of the *pufM* gene [1], [13]–[14] revealed high abundances and a heterogeneous distribution of AAPs world-wide [15]–[16]. It has been estimated that AAPs contribute up to 5% of the photosynthetic electron transport in oceanic surface waters [17].

Comparable to purple bacteria, AAPs are capable of light-driven and respiratory electron transport for their energy metabolism. However, this activity is different in several aspects. AAPs use light energy under oxic conditions and contain much less bacteriochlorophyll *a* (BChl *a*), with sometimes higher carotenoid than BChl a concentrations [18]. Increased amounts of carotenoids may possibly be a protective response against reactive oxygen species (ROS), which are produced in the light under oxic conditions [19]. As the biosynthesis of BChl *a* is known to be sensitive to ROS, AAPs synthesize BChl *a* only in the dark [20]–[21] and require a day and night cycle to sustain their photosynthetic activity. In accordance with the low BChl *content*, light is only used as a supplementary energy source [22]–[23]. Furthermore, AAPs are not autotrophs, but heterotrophs. Their photosynthetic activity is not used for CO_2_ fixation, but prevents the oxidation of organic substrates.

Light-driven proton translocation and ATP formation by AAPs have been investigated in several studies [24]–[25]. Recently, Tomasch et al. [26] studied the transcriptional response of *D. shibae* under different light regimes. The substrate-saving effect of light energy was demonstrated by lower respiration rates in the light [23], [25]. In chemostat cultures, the light-dependent increase of growth yields [27], [28] was reversely correlated to the growth rate and increased at low rates up to 110% [29].

The purpose of the present study was to identify the conditions under which AAPs benefit the most from their photosynthetic capacities. We hypothesized that AAPs benefit from photon energy under conditions of carbon and electron donor limitation, as proteomic responses to starvation and light conditions among AAPs have been reported [30]. Here, we performed long-term starvation experiments under different light regimes, and investigated survival and physiological fitness of *Dinoroseobacter shibae* [31] a representative of the globally abundant marine *Roseobacter* clade [32]–[34].

## Materials and Methods

### Organism and Media

*Dinoroseobacter shibae* strain DFL12^T^ was grown in both defined seawater and complex Marine Broth (Difco) medium. The seawater medium (SWM) contained (g per liter): NaCl, 20; Na_2_SO_4_, 4; MgCl_2_ * 6 H_2_O, 3; KCl, 0.5; NH_4_Cl, 0.25; KH_2_PO_4_, 0.2; CaCl_2_ * 2H_2_O, 0.15, NaHCO_3_, 0.19 g, 1 ml trace element solution SL12 [35] and 10 ml vitamin solution. The filter-sterilized vitamin solution contained 2 mg biotin, 20 mg nicotinic acid and 8 mg 4-aminobenzoic acid per liter. Marine Broth (MB) was used at 1/3 dilution. Furthermore, 10 mM sodium succinate was added to both media and the pH adjusted to 8.0. When solid medium was needed, 12 g of agar (Difco) was added per liter.

### Cultivation and Starvation Conditions

Batch cultures were incubated at 23°C on a shaker (Innova 42-R, New Brunswick, 125 rpm) in the dark (DD), under continuous illumination (LL; 12 µmol photons m^−2^ s^−1^) or light and dark cycles (LD, 12 h/12 h, 12 µmol photons m^−2^ s^−1^). For determining the optimum light intensity, GRO-LUX fluorescent light bulbs were used as light source. Growth was monitored by measuring the optical density (OD) at 650 nm. For starvation experiments, stationary phase cells were harvested by centrifugation at 3330×*g* in a Sorvall RC-2 refrigerated centrifuge for 15 min at 5°C and washed with salt solution containing 20 g NaCl and 0.5 g KCl per liter. Cells were resuspended in salt solution (sea water medium without carbon source 250 ml in 500 ml sterile Erlenmeyer flasks) and incubated as described above.

### Cell Counts and Viability

Total cell counts were analyzed by SYBR Green staining and epifluorescence microscopy (Olympus BX51). For live counts, serially diluted samples were plated onto MB agar plates incubated at 25°C and counted after 4–8 weeks. To study morphological changes under different conditions, suspensions of starved cells were placed on a Formvar copper grid (*Plano*) for 5 min. Adsorbed cells were stained with 0.5% aqueous uranyl acetate for 1 min followed by a distilled water rinse and examined with a Zeiss EM 902A transmission electron microscope. A Proscan High Speed SSCCD camera system with iTEMfive software was used for image acquisition. At least fifty pictures were analyzed to compare changes in cell morphology.

### Estimation of Dry Biomass, Protein Content, PHA and Photosynthetic Pigments

To determine the dry biomass, starved cells were harvested and washed with 50 mM ammonium acetate buffer and dried overnight at 80°C. Lowry’s method with Folins reagent was used to determine the total protein concentration [36]. The *in vivo* photo pigments were analyzed by recording the absorption spectrum of whole cells in a UV/VIS spectrophotometer (Perkin Elmer, Lambda 2S) with a resolution of 1 nm from 350 to 950 nm. Polyhydroxyalkanoate (PHA) content was determined using Nile blue staining [37]. For pigment analysis, cells were centrifuged at 6000 *g* for 20 min and pigments were extracted from the pellet with 1 ml acetone:methanol (7∶2) for 1 hour in the dark. BChl *a* absorption was determined at 772 nm with an extinction coefficient of 75 mM^−1^ cm^−1^ [38]. Carotenoids were quantified at 482 nm using an extinction coefficient of 123.6 mM^−1^ cm^−1^ [39].

### Determination of Respiration Rates

Washed cells were resuspended in HEPES buffer (10 mM, pH 7.75, supplemented with NaCl 20 g per liter, KCl 0.5 g per liter). Oxygen concentrations were measured with a Clark-type oxygen electrode (Bachofer, Reutlingen, Germany) while the chamber was maintained at 30°C. The influence of light on the respiration was checked by illuminating the cells in the reaction chamber using a halogen lamp at 400 µmol photons m^−2^ s^−1^.

### Determination of ATP

Washed cell suspensions (3 ml) were incubated in Hungate tubes sealed with rubber stoppers and flushed with nitrogen at room temperature. The physiological responses were analysed by incubation of cell suspensions under anoxic conditions and studying the reaction upon oxygen and organic substrate addition in the dark and in the light. The energy content of the cells was determined using ATP bioluminescence Assay Kit CLS II (Boehringer Mannheim) and the extraction of ATP was carried out as described in [25].

### Proton Translocation Measurements

Washed cells were resuspended in 2.5 ml salt solution containing 2% NaCl and 0.05% KCl and treated with 500 µl of 0.5 M KSCN, thus providing the membrane-permeable anion SCN^–^ in order to destroy the membrane potential. The cell suspensions were then flushed with nitrogen for 20 min. Known amounts of oxygen (16 nmol O_2_) in KCl solution, were injected into the measuring chamber equipped a with pH electrode (Mettler Toledo pH Electrode, Inlab Micro).

## Results

### Optimum Light Intensity for Starvation Experiments

Cells pre-grown with succinate at different light intensities were harvested, washed, resuspended in medium without carbon source, and incubated under light and dark cycles (LD, 12 h/12 h) for four weeks. Different light intensities were tested to determine the optimum illumination for survival. Under all conditions, total and viable cell counts decreased However, cultures incubated at medium light intensity (12 µmol photons m^−2^ s^−1^) showed higher survival rates (Table 1), reaching tenfold higher cell counts than those incubated at high (23 µmol photons m^−2^ s^−1^) or low (3 µmol photons m^−2^ s^−1^) light intensities. Protein to biomass ratios did not show any significant differences between the light conditions. In contrast, at medium and low light intensities carotenoid concentrations decreased whereas bacteriochlorophyll a concentrations remained constant. At high light intensity, bacteriochlorophyll a concentrations decreased, while carotenoid concentrations increased by a factor of 5 indicating their photo-protective function.

**Table 1:** Biomass parameters of *Dinoroseobacter shibae* upon starvation.

		Light intensities (µmol m^−2^ s^−1^)		
Days starved	Parameters	High	Middle	Low
		23	12	3
0	Protein/biomass	0.41±0.02	0.46±0.02	0.49±0.01
	Total cell counts/ml (10^8^)	2.62±3	2.6±2	2.66±1.3
	Viable counts/ml (10^8^)	1.97±1.2	2.28±1.4	2.10±0.18
	BChl *a* (nmol/mg protein)	2.12±2	2.82±3	2.7±2
	Carotenoids (nmol/mg protein)	1.39±0.03	1.88±0.07	1.87±0.11
14	Protein/biomass	0.36±0.05	0.38±0.02	0.36±0.02
	Total cell counts/ml (10^8^)	2.17±0.85	2.36±1.95	1.46±2
	Viable counts/ml (10^7^)	1.91±0.11	16.3±0.23	2.67±3.1
	BChl *a* (nmol/mg protein)	1.11±3	1.96±1	1.66±5
	Carotenoids (nmol/mg protein)	1.17±0.03	1.26±0.52	1.11±0.05
21	Protein/biomass	0.33±0.03	0.33±0.01	0.24±0.1
	Total cell counts/ml (10^8^)	1.06^a^	2.14±0.08	1.31±2
	Viable counts/ml (10^6^)	10.4^a^	117.21±1.83	6.27±0.02
	BChl *a* (nmol/mg protein)	0.68±3	1.17±1	1.06±5
	Carotenoids (nmol/mg protein)	0.76±0.11	0.61±0.03	0.42±0.07

^a^ Cell counts were performed from single bottle as the biological replicate cells started to clump.

### Morphological and Physiological Adaptations upon Nutrient Limitation

**Figure 1:**
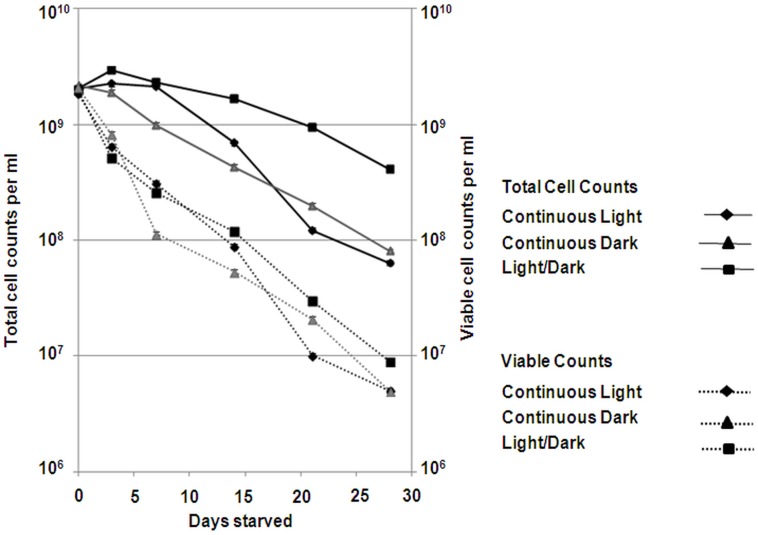
Changes in total and viable counts of *D. shibae* during starvation. Symbols: total (solid lines) and viable (broken lines) counts of cells starved under light/dark cycles (LD, 12/12 h, squares), continuous light (diamonds), or in the dark (triangles). The light intensity was 12 µmol photons m^−2^ s^−1^. Mean values and standard deviation shown are from two biological replicates.

**Figure 2:**
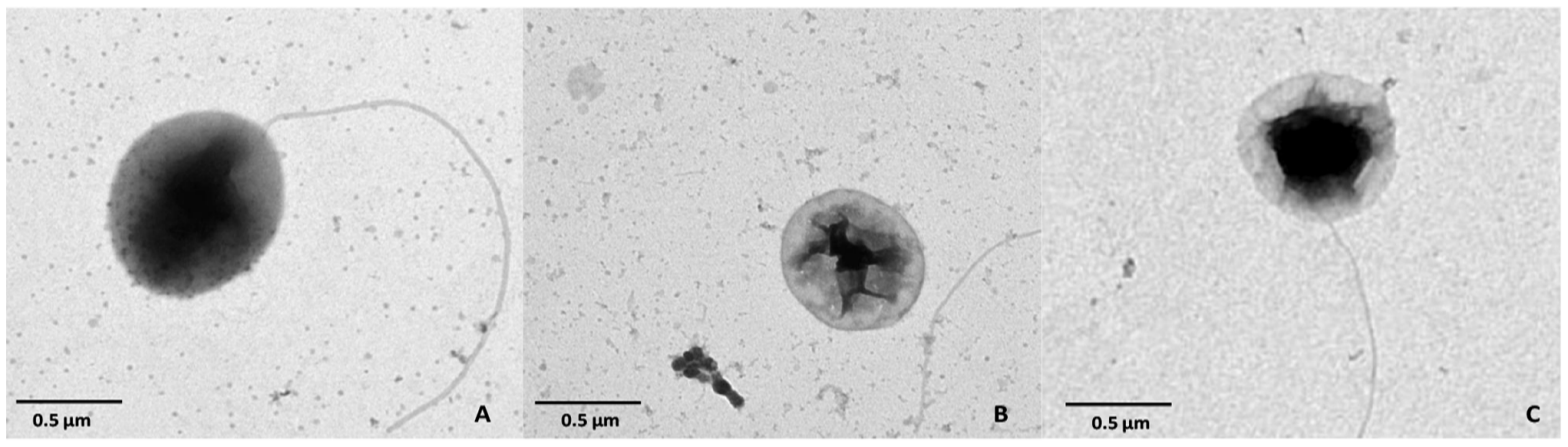
Morphological changes analysed by transmission electron microscopy. (A) Freshly grown cells of *D.shibae* under light and dark cycle (LD, 12/12 h) were regular shaped and flagellated. (B) Cells starved in the dark (DD) were wrinkled and not flagellated, whereas (C) some LD cells still possessed flagella after three weeks of starvation.

Starvation experiments were performed at the optimum light intensity (12 µmol photons m^−2^ s^−1^) under different light regimes, i.e. light and dark cycle (LD), continuous light (LL), and continuous dark (DD). LD conditions resulted in the highest survival rates. During the first week of starvation, total and viable cell counts of the DD cultures decreased faster than those in LL and LD cultures (Figure 1). After four weeks of starvation, LD cultures resulted in 10-fold higher total and viable cell counts. Cell morphology was affected both in LL and DD cultures, as cells appeared irregular and wrinkled and flagella were detached anymore (Figure 2). In contrast, cells in LD cultures had a less irregular shape, and some cells still carried flagella after four weeks. Nile-blue staining showed the presence of polyhydroxyalkanotes (PHA) during the first days of starvation (Figure S1). After four weeks, PHAs disappeared in LL and DD cells, but were still visible in LD cells.

The physiological fitness of cells was assessed by respiration rates, respiration-driven proton translocation, and the ability of ATP regeneration. Respiration rates served as a measure of physiological activity and light utilization. The slowdown of respiration in the light indicates saving of electron donors and light-driven energy conservation. Independent of the starvation conditions, endogenous respiration rates were 10% higher in the dark than under illumination during the early starvation phase. Addition of succinate as electron donor resulted in a similar effect. After 4 weeks of starvation only LD cells showed this effect (Table 2).

**Table 2:** Endogenous and succinate-dependent respiration rates in *Dinoroseobacter shibae* after starvation.

Starvation conditions	Days starved	Endogenous respiration (nmol O_2_ min^−1^ (mg DW) ^−1^)		
		Dark	Light	Dark again
**LL**	3	23.78±1.2	20.79±2.3	21.89±0.9
	7	15.66±0.91	14.09±1.3	15.23±2
	21^a^	2.09	2.09	2.09
**LD**	3	22.13±4	18.77±1.2	20.63±0.6
	7	17.57±2.1	16.35±0.83	17.01±1.9
	21	9.31±0.38	9.24±2	9.21±2
		**Substrate-specific respiration (nmol O_2_ min^−1^ (mg DW) ^−1^)**		
		**Dark**	**Light**	**Dark again**
**LL**	3	27.63±4.3	25.01±2.8	25.83±0. 5
	7	19.87±2.5	17.21±0.68	18.19±0.41
	21^a^	4.75	4.75	4.75
**LD**	3	22.68±2.8	22.06±3.1	21.33±2.6
	7	18.59±1.8	17.96±1	18.12±1
	21	9.99±0.95	9.19±1.1	9.39±0.2

[Rates were measured in the dark, after switching on light (400 µmol photons m^−2^ s^−1^) for 2 min, and in the dark again].

^a^ Respiration rates were performed from single bottle as the other harvested cells started to clump after anoxygenic incubation.

**Figure 3:**
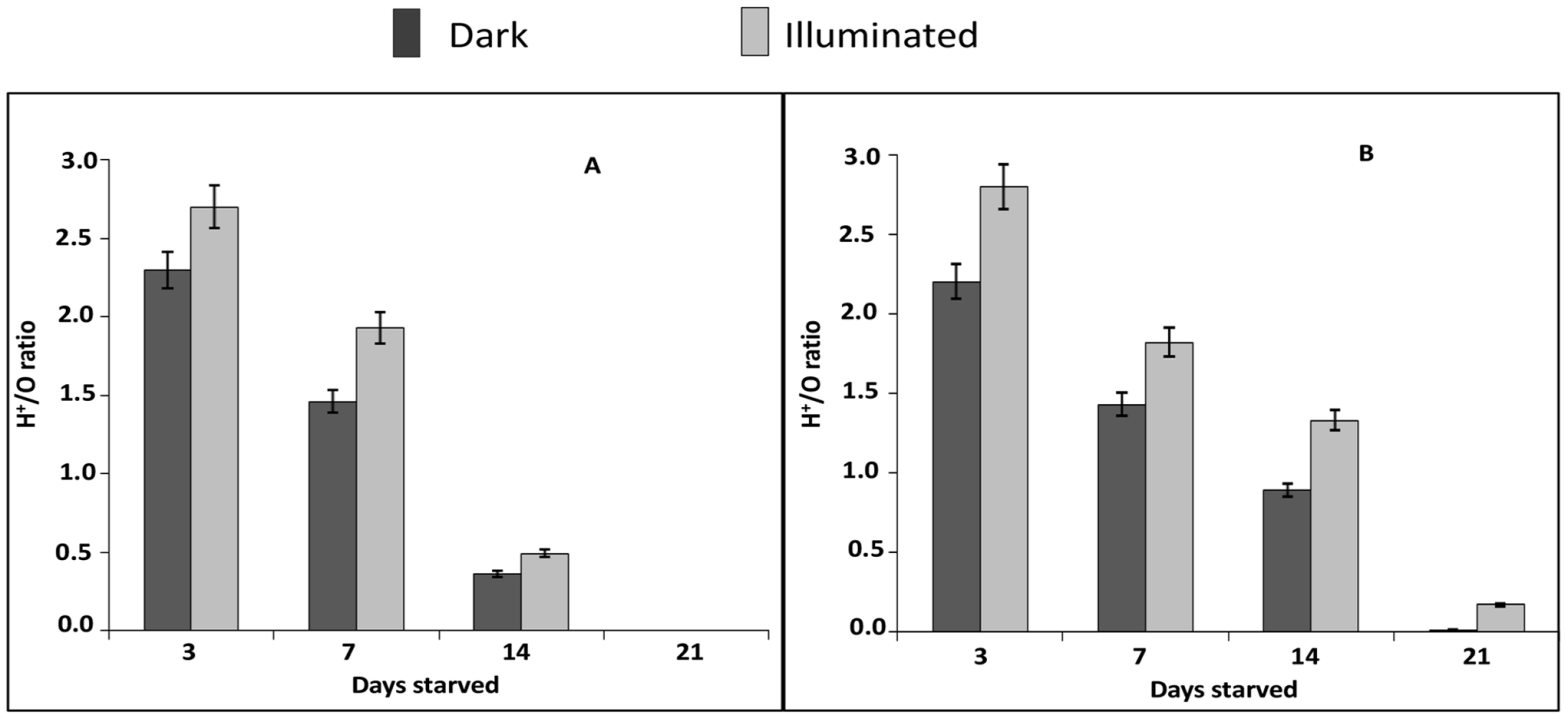
Respiration- and light-driven proton translocation in *D. shibae*. Cells were maintained in the dark (dark bar) and the influence of light on the number of translocated per added oxygen atom (H^+^/O) were studied when cells were illuminated (grey bar). (A) Cells starved under continuous light (LL); (B) cells starved under light and dark cyclers (LD). Light intensity: 400 µmol photons m^−2^ s^−1^. Mean values and standard deviation shown are from two biological replicates.

Utilization of light was also shown in proton translocation measurements, when oxygen and light were supplied simultaneously to cells incubated under N_2_ (Figure 3 a and b). The highest ratio of translocated protons per added oxygen atom (H^+^/O) in the dark was 2.3, independent of the starvation conditions. When cell suspensions were additionally illuminated and charged with oxygen pulses (16 nmol), the H^+^/O ratios increased to 2.8. After two weeks of starvation, H^+^/O ratios of LL cells had dropped by a factor of 4, while only decreasing by half in LD cells.

The cytoplasmic ATP concentrations showed a steady decrease upon starvation (Table 3). Initial ATP concentrations were 3 * 10^−18^ mol ATP per cell, decreasing by 90% in both LL and LD cells after 3 weeks. When the cells were washed and incubated anoxically, the ATP levels decreased close to zero within 2 hours. With the addition of oxygen and light, ATP was regenerated by more than 50% within two minutes.

**Table 3:** Cytoplasmic ATP concentrations in cell suspensions of *D. shibae starved* under LL and LD conditions.

	(10^−18^) mol ATP per cell			(10^−18^) mol ATP per cell		
Days starved	LL	LL+N_2_	O_2_+ light	LD	LD+N_2_	O_2_+ light
3	3.02±0.2	0.01±0.2	2.66±0.2	2.95±0.2	0.58±0.009	2.25±0.2
7	1.9±0.1	0.03±0.0	1.47±0.06	2.21±0.2	0.005±0.02	1.71±0.02
14	0.91±0.1	0.05±0.1	0.61±0.02	1.09±0.01	0.0006±0.01	0.9±0.04
21	0.22±0.2	0.01±0.0	0.09±0.1	0.45±0.04	0.004±0.001	0.21±0.01

(ATP was measured after harvesting the cells, after anoxic incubation for 2 h, and after flushing the suspension with air in the light [400 µmol photons m^−2^ s^−1^, 2 min].

### Survival of Cells Grown in Complex Compared to Defined Medium

Under DD conditions, total cell counts in complex media were stable over six weeks (Figure S2), whereas with succinate they decreased more than tenfold after 1 month. Unfortunately, the light intensity for LD and LL conditions was high (53 µmol photons m^−2^ s^−1^) since these experiments were carried out prior to the determination of the optimum light intensities. Here, cell counts declined faster under LD conditions than in the dark, which underlines the detrimental effects of high light intensity (Table S1, Table S2 and Table S3).

## Discussion

Our study demonstrated that day and night cycle substantially increases the survival of *Dinoroseobacter shibae* during long term starvation. However, high light intensity may be detrimental and induce protective reactions.

### The Diurnal Light and Dark Regime Increases Cell Fitness

All measured parameters show that *Dinoroseobacter shibae* benefits from exposure to a diurnal light and dark regime. Under this condition, the cells survived starvation with better physiological fitness. As cells were shown to preserve intracellular PHA, known to be the main storage material in *D. shibae* [28], cells maintained increased dry mass to protein ratios. Cell pigmentation, morphology and motility of cells grown under the diurnal light cycle were less affected than those in the dark or under continuous light. Viability and fitness of cells decreased in similar manner both in the dark and under continuous light, which implies that light inhibits BChl a synthesis and prevents photosynthetic activity over longer periods. The increased fitness of LD-starved cells was also found by quantifying respiration, proton translocation and ATP regeneration. The beneficial effects became more pronounced with prolonged starvation. These observations could be explained by one underlying rationale: Light utilization appears rather as a mechanism to survive starvation than as a growth-promoting factor, which improves survival and physiological fitness several-fold.

### Light as a Stress Factor

Not only continuous light, but also high intensities during day and night cycles had negative effects on the cells. The harmful light effects were also recognized from the pigment analysis, as cells increased their carotenoid contents at high light intensities likely as a protective response. By low pigment concentrations, AAPs might prevent formation of reactive oxygen species [19]. This was corroborated by the low optimum light intensity of 12 µmol photons m^−2^ s^−1^, which might additionally minimize ROS exposure. The optimum light intensity of 12 µmol photons m^−2^ s^−1^ is much lower than mid-day intensities in, e.g., the North Pacific Gyre, which average 50–150 µmol photons m^−2^ s^−1^ at 20–150 m water depth [40]. Thus, low-light adaption of *Dinoroseobacter shibae* minimizes ROS exposure and might explain why none of the AAPs is able to grow purely phototrophic.

### Ecological Implications

The light-supported survival of starvation of *D.shibae* has also been reported for other groups of bacteria. In a bacteriochlorophyll *a*-containing gammaproteobacterium, expression of the photosynthesis genes depended on the type of carbon source [41]. Also some proteorhodopsin-carrying bacteria such as the abundant Cand. *Pelagibacter ubique* [42], *Dokdonia* sp. strain MED134 [43] and *Vibrio* sp. strain AND4 [44], benefit from light during starvation.

Considering the specific advantage of the AAPs in their natural environment, it becomes clear that light is not the overall limiting factor for their distribution. Cottrell et al. found that AAPs are distributed over the whole photic zone [40]. In a particle-rich estuary, the BChl a concentrations of AAPs varied in response to particles but not to light limitation [45]. In a recent study, Čuperová et al. showed that the DOC concentrations influenced the AAP abundance in alpine lakes [46]. In agreement with these findings, our study suggests that the limitation of organic substrates might promote the competitiveness of AAPs. Nutrient limitation is a predominating feature in most oceanic regions. Biomass of AAPs in the South Pacific Ocean was on average two-fold higher than that of other prokaryotic cells [11], [16]. Obviously, AAPs can profit from light utilization by conserving instead of oxidizing organic substrates or their storage compounds.

## Supporting Information

Figure S1 **Detection of polyhydroxyalkanoate (PHA) in** ***D. shibae*** **by Nile blue staining.** Cells starved under (A) early stationary phase and (B) after three weeks of starvation under light and dark cycle (LD) conditions.
(PPT)

Figure S2 **Changes in total and viable counts of** ***D. shibae*** **during starvation in complex medium.** Symbols: total (solid lines) and viable (broken lines) counts of cells starved under light/dark cycles (LD, 12/12 h, squares, 53 µmol photons m^−2^ s^−1^), continuous light (diamonds, 26 µmol photons m^−2^ s^−1^), or in the dark (triangles).
(PPT)

Table S1 **Protein/biomass and bacteriochlorophyll a concentrations of** ***Dinoroseobacter shibae*** **upon starvation under complex media.**
(PPT)

Table S2 **ATP concentrations of** ***Dinoroseobacter shibae*** **upon starvation under complex media.** ATP was measured after harvesting the cells, after anoxic incubation for 2 h, and after flushing the suspension with air in the light [400 µE m^−2^ s^1^, 2 min] for 2 min.
(PPT)

Table S3 **Endogenous and substrate specific respiration rates of** ***Dinoroseobacter shibae*** **upon starvation under complex media.** Rates were measured in the dark, after switching on light (400 µE m^−2^ s^1^) for 2 min, and in the dark again.
(PPT)
