# Supplementary material for: Light Enhances Survival of Dinoroseobacter shibae during Long-Term Starvation
Source: PLoS One. 2013 Dec 30;8(12):e83960. doi: 10.1371/journal.pone.0083960 (PMC3875502; doi:10.1371/journal.pone.0083960)
Supplement: Figure S1 — Detection of polyhydroxyalkanoate (PHA) in D. shibae by Nile blue staining. Cells starved under (A) early stationary phase and (B) after three weeks of starvation under light and dark cycle (LD) conditions. (PPT) [file pone.0083960.s001.ppt]

## Slide 1
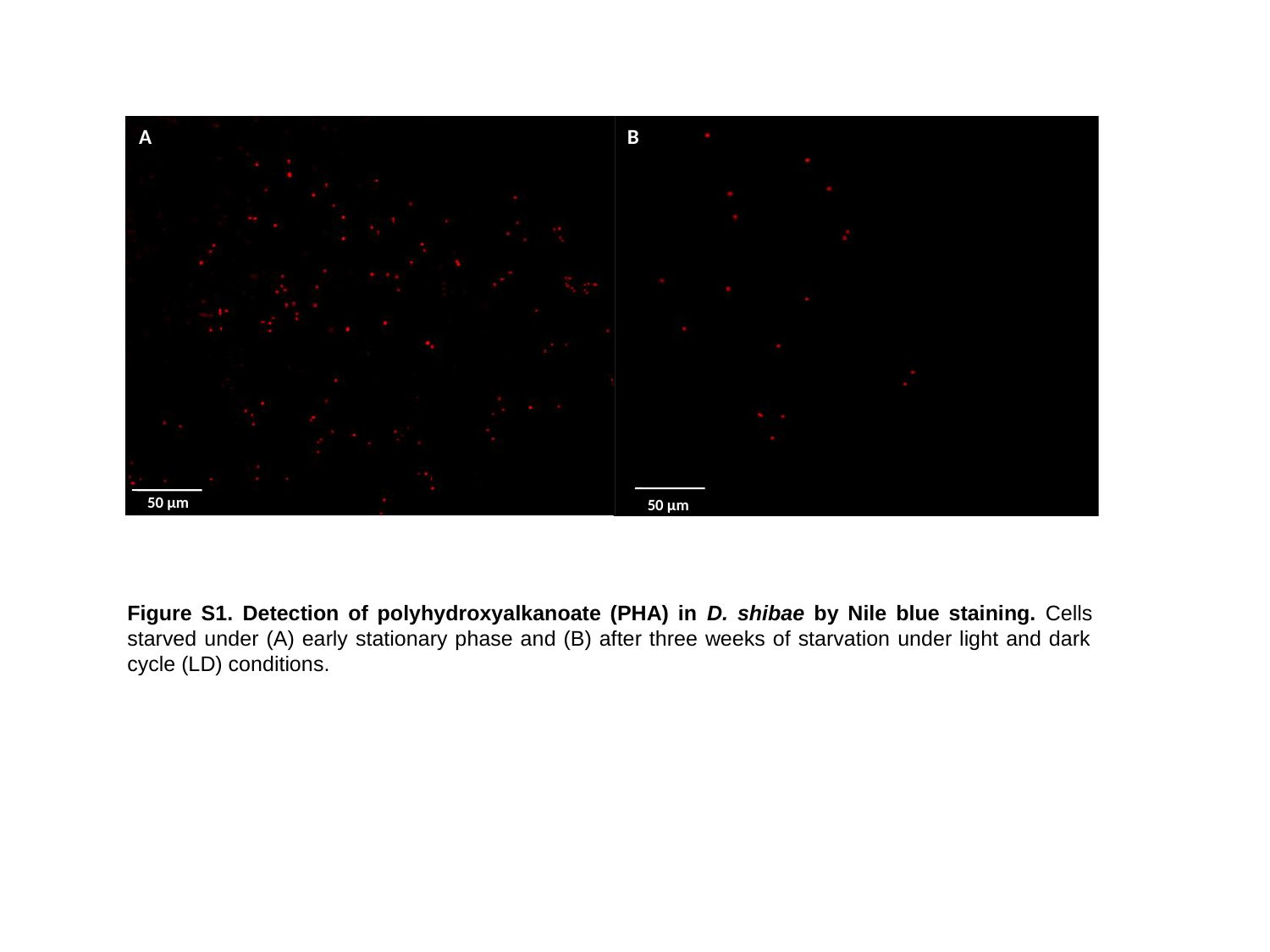

50 µm
50 µm
A
B
Figure S1. Detection of polyhydroxyalkanoate (PHA) in D. shibae by Nile blue staining. Cells starved under (A) early stationary phase and (B) after three weeks of starvation under light and dark cycle (LD) conditions.
