# Supplementary material for: Light Enhances Survival of Dinoroseobacter shibae during Long-Term Starvation
Source: PLoS One. 2013 Dec 30;8(12):e83960. doi: 10.1371/journal.pone.0083960 (PMC3875502; doi:10.1371/journal.pone.0083960)
Supplement: Figure S2 — Changes in total and viable counts of D. shibae during starvation in complex medium. Symbols: total (solid lines) and viable (broken lines) counts of cells starved under light/dark cycles (LD, 12/12 h, squares, 53 µmol photons m−2 s−1), continuous light (diamonds, 26 µmol photons m−2 s−1), or in the dark (triangles). (PPT) [file pone.0083960.s002.ppt]

## Slide 1
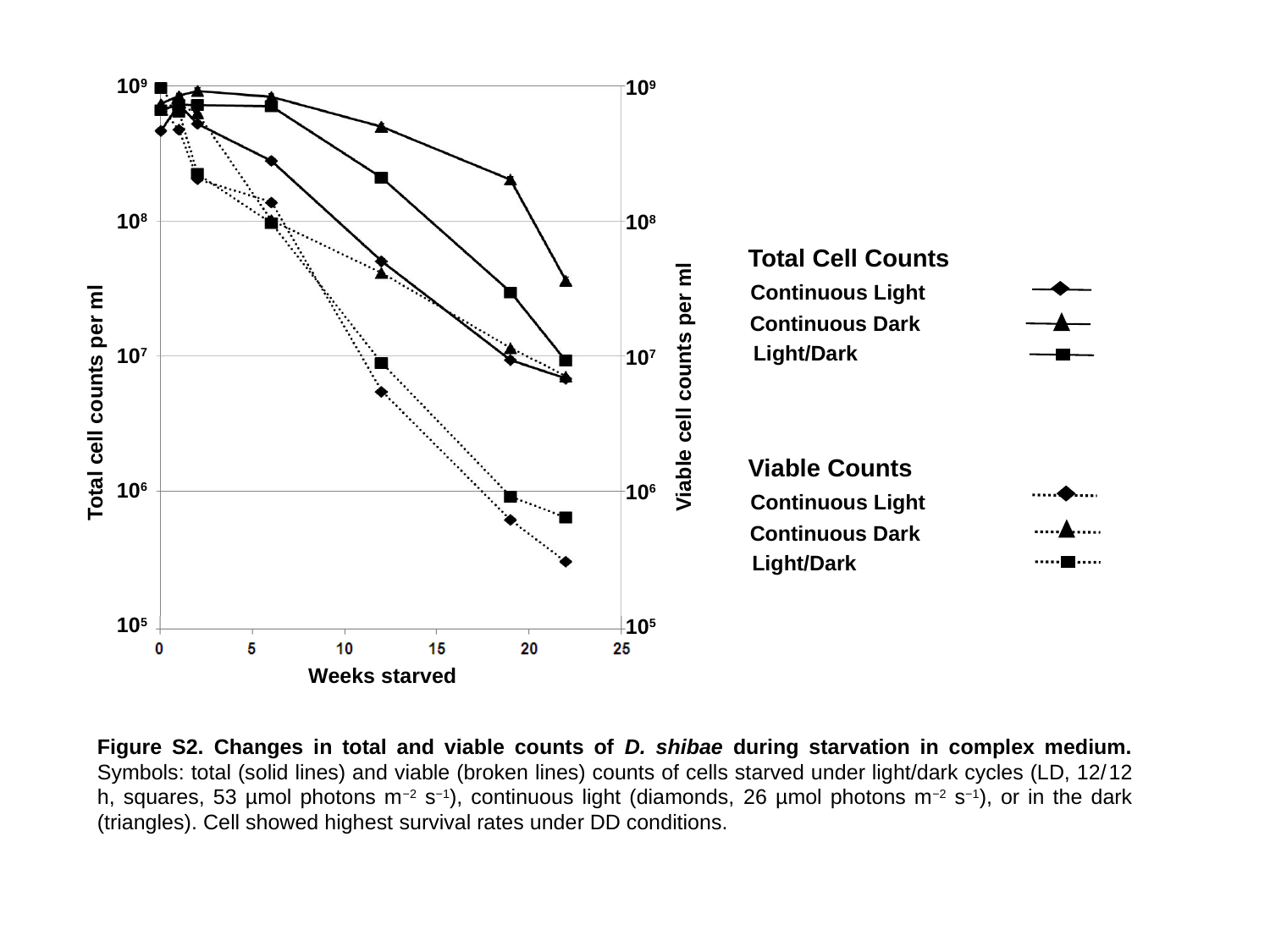

109
108
107
106
105
109
108
107
106
105
Total Cell Counts
Continuous Light
Continuous Dark
Light/Dark
Viable Counts
Continuous Light
Continuous Dark
Light/Dark
Viable cell counts per ml
Total cell counts per ml
Weeks starved
Figure S2. Changes in total and viable counts of D. shibae during starvation in complex medium. Symbols: total (solid lines) and viable (broken lines) counts of cells starved under light/dark cycles (LD, 12/12 h, squares, 53 µmol photons m−2 s−1), continuous light (diamonds, 26 µmol photons m−2 s−1), or in the dark (triangles). Cell showed highest survival rates under DD conditions.
