# Supplementary material for: Light Enhances Survival of Dinoroseobacter shibae during Long-Term Starvation
Source: PLoS One. 2013 Dec 30;8(12):e83960. doi: 10.1371/journal.pone.0083960 (PMC3875502; doi:10.1371/journal.pone.0083960)
Supplement: Table S1 — Protein/biomass and bacteriochlorophyll a concentrations of Dinoroseobacter shibae upon starvation under complex media. (PPT) [file pone.0083960.s003.ppt]

## Slide 1
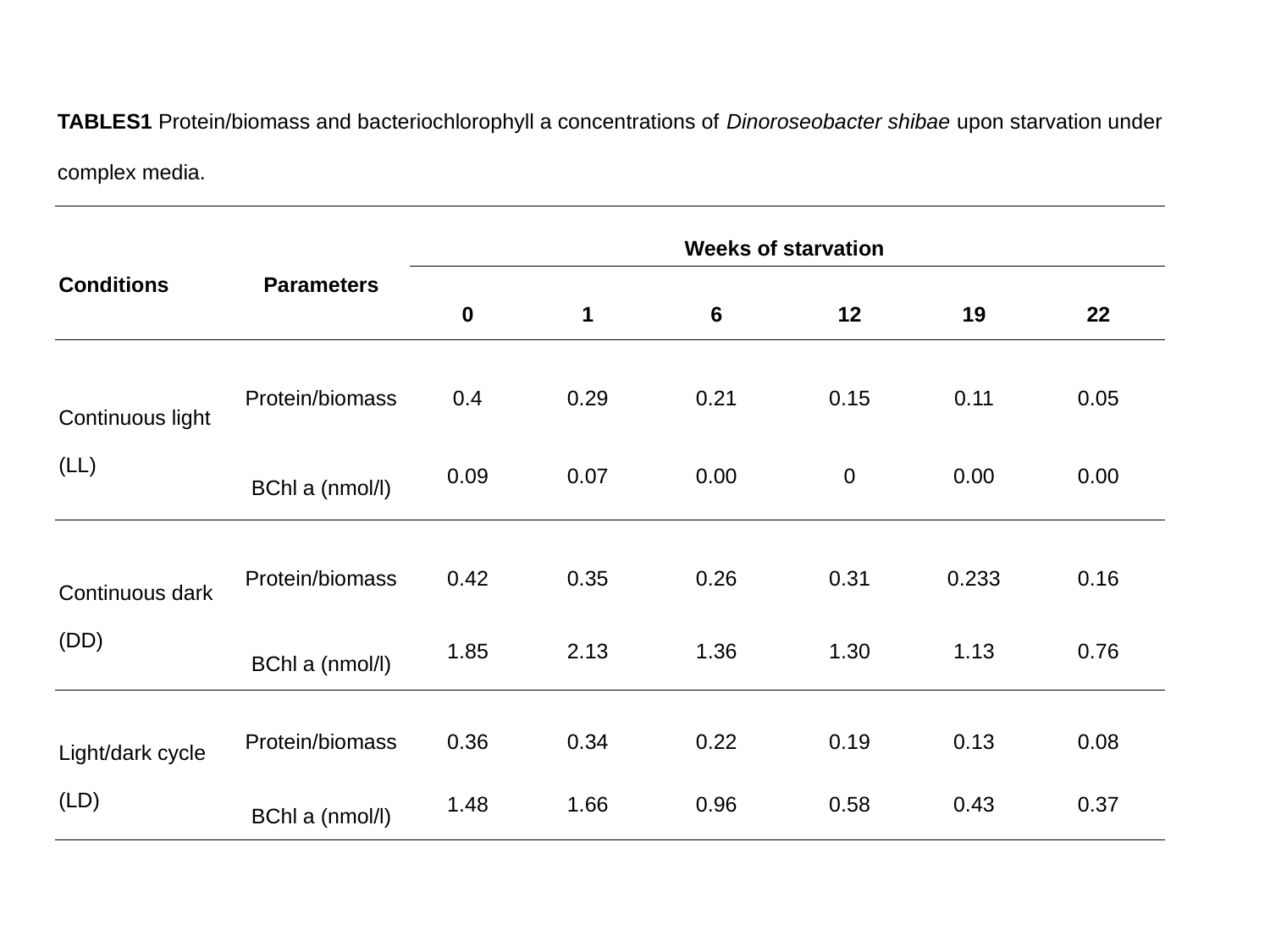

TABLES1 Protein/biomass and bacteriochlorophyll a concentrations of Dinoroseobacter shibae upon starvation under complex media.
| Conditions | Parameters | Weeks of starvation | | | | | |
| --- | --- | --- | --- | --- | --- | --- | --- |
| | | 0 | 1 | 6 | 12 | 19 | 22 |
| Continuous light (LL) | Protein/biomass | 0.4 | 0.29 | 0.21 | 0.15 | 0.11 | 0.05 |
| | BChl a (nmol/l) | 0.09 | 0.07 | 0.00 | 0 | 0.00 | 0.00 |
| Continuous dark (DD) | Protein/biomass | 0.42 | 0.35 | 0.26 | 0.31 | 0.233 | 0.16 |
| | BChl a (nmol/l) | 1.85 | 2.13 | 1.36 | 1.30 | 1.13 | 0.76 |
| Light/dark cycle (LD) | Protein/biomass | 0.36 | 0.34 | 0.22 | 0.19 | 0.13 | 0.08 |
| | BChl a (nmol/l) | 1.48 | 1.66 | 0.96 | 0.58 | 0.43 | 0.37 |
