# Supplementary material for: Light Enhances Survival of Dinoroseobacter shibae during Long-Term Starvation
Source: PLoS One. 2013 Dec 30;8(12):e83960. doi: 10.1371/journal.pone.0083960 (PMC3875502; doi:10.1371/journal.pone.0083960)
Supplement: Table S2 — ATP concentrations of Dinoroseobacter shibae upon starvation under complex media. ATP was measured after harvesting the cells, after anoxic incubation for 2 h, and after flushing the suspension with air in the light [400 µE m−2 s1, 2 min] for 2 min. (PPT) [file pone.0083960.s004.ppt]

## Slide 1
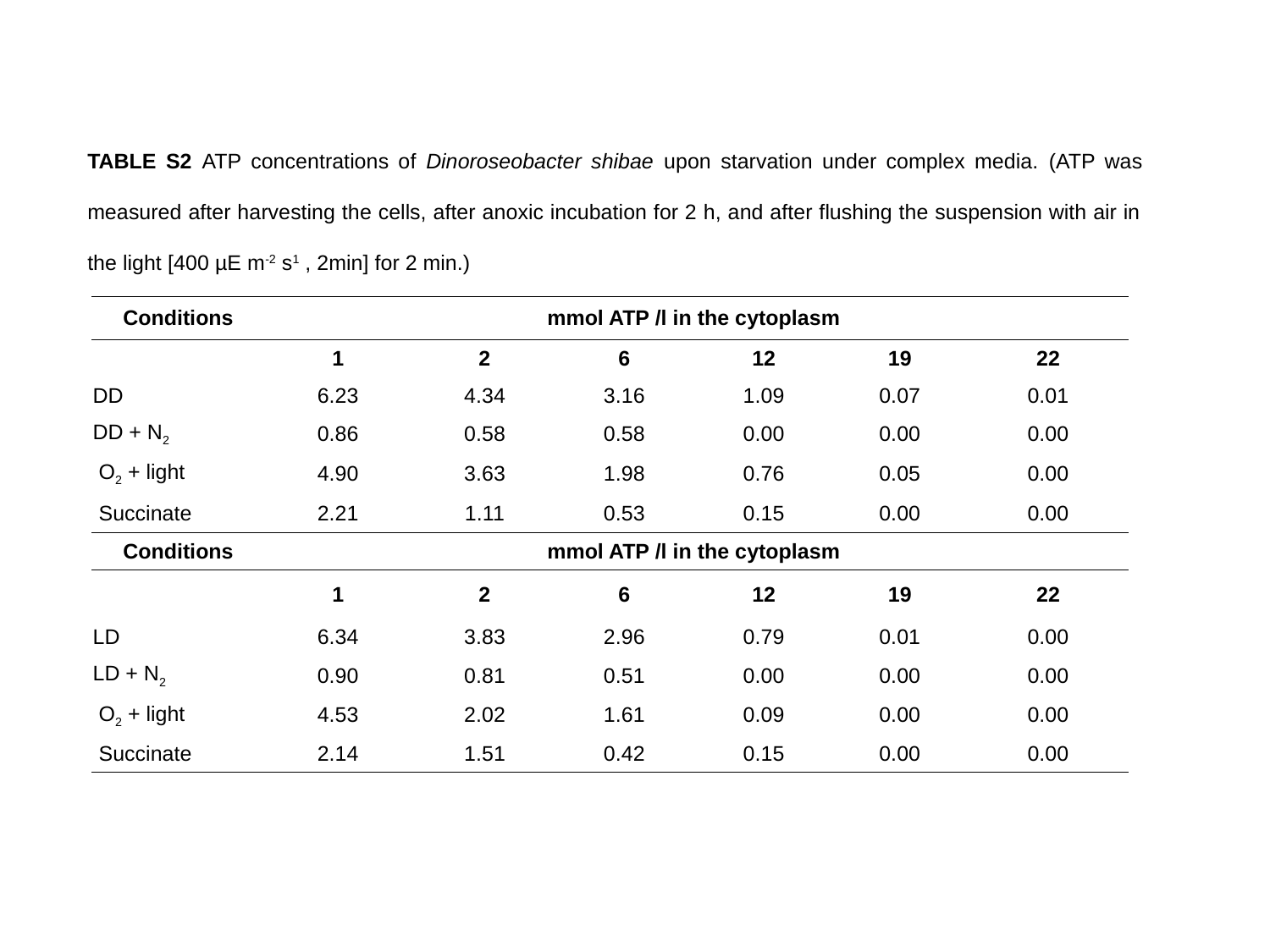

TABLE S2 ATP concentrations of Dinoroseobacter shibae upon starvation under complex media. (ATP was measured after harvesting the cells, after anoxic incubation for 2 h, and after flushing the suspension with air in the light [400 µE m-2 s1 , 2min] for 2 min.)
| Conditions | mmol ATP /l in the cytoplasm | | | | | |
| --- | --- | --- | --- | --- | --- | --- |
| | 1 | 2 | 6 | 12 | 19 | 22 |
| DD | 6.23 | 4.34 | 3.16 | 1.09 | 0.07 | 0.01 |
| DD + N2 | 0.86 | 0.58 | 0.58 | 0.00 | 0.00 | 0.00 |
| O2 + light | 4.90 | 3.63 | 1.98 | 0.76 | 0.05 | 0.00 |
| Succinate | 2.21 | 1.11 | 0.53 | 0.15 | 0.00 | 0.00 |
| Conditions | mmol ATP /l in the cytoplasm | | | | | |
| | 1 | 2 | 6 | 12 | 19 | 22 |
| LD | 6.34 | 3.83 | 2.96 | 0.79 | 0.01 | 0.00 |
| LD + N2 | 0.90 | 0.81 | 0.51 | 0.00 | 0.00 | 0.00 |
| O2 + light | 4.53 | 2.02 | 1.61 | 0.09 | 0.00 | 0.00 |
| Succinate | 2.14 | 1.51 | 0.42 | 0.15 | 0.00 | 0.00 |
