# Supplementary material for: Light Enhances Survival of Dinoroseobacter shibae during Long-Term Starvation
Source: PLoS One. 2013 Dec 30;8(12):e83960. doi: 10.1371/journal.pone.0083960 (PMC3875502; doi:10.1371/journal.pone.0083960)
Supplement: Table S3 — Endogenous and substrate specific respiration rates of Dinoroseobacter shibae upon starvation under complex media. Rates were measured in the dark, after switching on light (400 µE m−2 s1) for 2 min, and in the dark again. (PPT) [file pone.0083960.s005.ppt]

## Slide 1
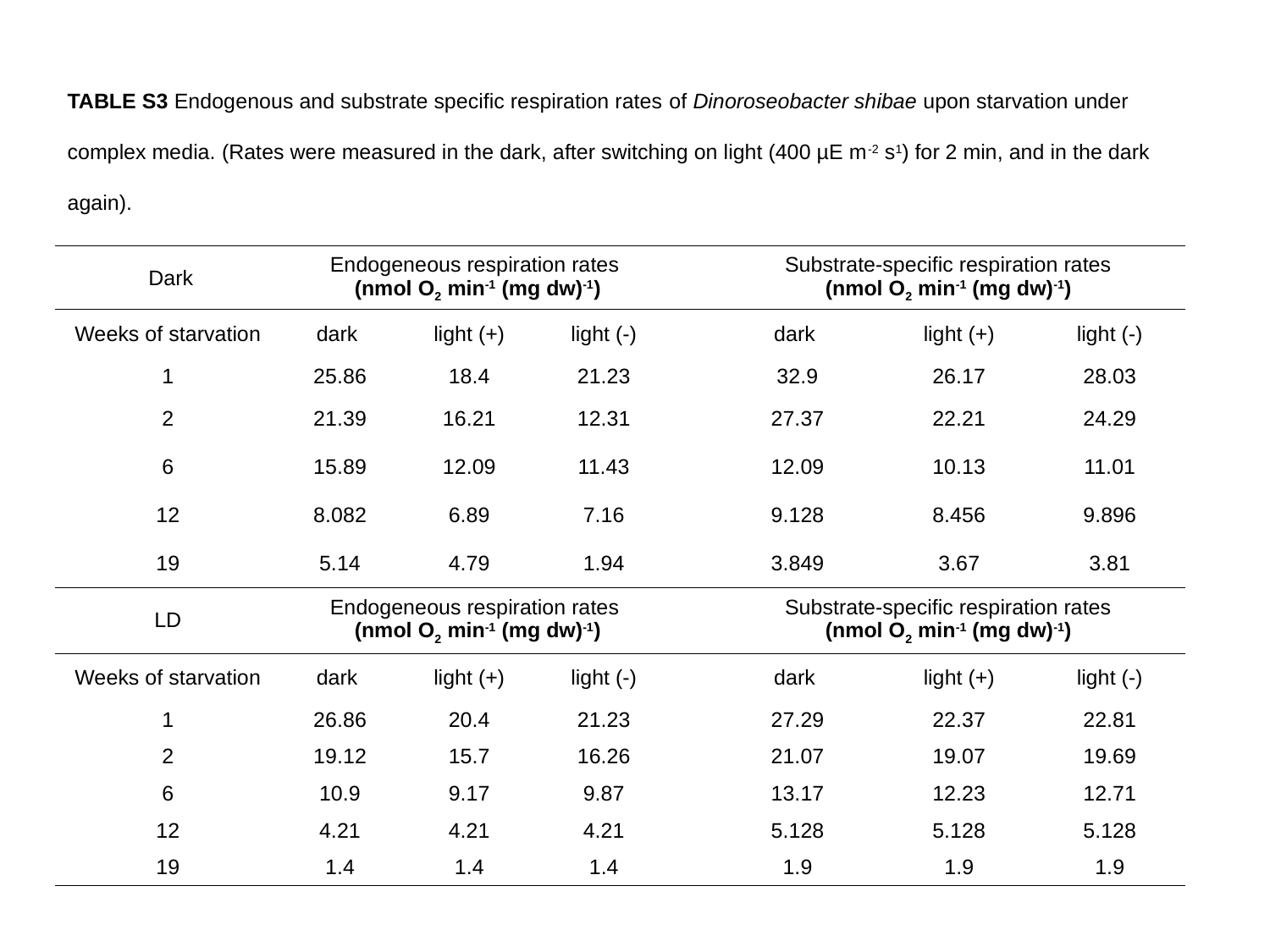

TABLE S3 Endogenous and substrate specific respiration rates of Dinoroseobacter shibae upon starvation under complex media. (Rates were measured in the dark, after switching on light (400 µE m-2 s1) for 2 min, and in the dark again).
| Dark | Endogeneous respiration rates (nmol O2 min-1 (mg dw)-1) | | | | Substrate-specific respiration rates (nmol O2 min-1 (mg dw)-1) | | |
| --- | --- | --- | --- | --- | --- | --- | --- |
| Weeks of starvation | dark | light (+) | light (-) | | dark | light (+) | light (-) |
| 1 | 25.86 | 18.4 | 21.23 | | 32.9 | 26.17 | 28.03 |
| 2 | 21.39 | 16.21 | 12.31 | | 27.37 | 22.21 | 24.29 |
| 6 | 15.89 | 12.09 | 11.43 | | 12.09 | 10.13 | 11.01 |
| 12 | 8.082 | 6.89 | 7.16 | | 9.128 | 8.456 | 9.896 |
| 19 | 5.14 | 4.79 | 1.94 | | 3.849 | 3.67 | 3.81 |
| LD | Endogeneous respiration rates (nmol O2 min-1 (mg dw)-1) | | | | Substrate-specific respiration rates (nmol O2 min-1 (mg dw)-1) | | |
| Weeks of starvation | dark | light (+) | light (-) | | dark | light (+) | light (-) |
| 1 | 26.86 | 20.4 | 21.23 | | 27.29 | 22.37 | 22.81 |
| 2 | 19.12 | 15.7 | 16.26 | | 21.07 | 19.07 | 19.69 |
| 6 | 10.9 | 9.17 | 9.87 | | 13.17 | 12.23 | 12.71 |
| 12 | 4.21 | 4.21 | 4.21 | | 5.128 | 5.128 | 5.128 |
| 19 | 1.4 | 1.4 | 1.4 | | 1.9 | 1.9 | 1.9 |
